# Comparative evaluation of CD34 immunohistochemistry and hematoxylin and eosin staining for detection of lymphovascular emboli in oral squamous cell carcinoma: a retrospective cross-sectional study in a developing country

**DOI:** 10.1007/s12672-025-03633-6

**Published:** 2025-09-30

**Authors:** Kajal Hatgaonkar, Anand Hatgaonkar, Pratibha Dawande, Shweta Sonkusale, Shruti Deshpande

**Affiliations:** 1 Department of Pathology, Datta Meghe Medical College, DMIHER, Wardha, Nagpur, India; 2 Department of Radiodiagnosis, Datta Meghe Medical College, DMIHER, Wardha, Nagpur, India

## Abstract

**Objectives:**

The primary objective was to evaluate and compare the sensitivity and specificity of CD34 immunohistochemistry (IHC) versus conventional hematoxylin and eosin (H&E) staining in detecting lymphovascular emboli (LVE) in oral squamous cell carcinoma (OSCC). The secondary objective was to correlate LVE detection with tumor size, grade, perineural invasion (PNI), depth of invasion (DOI), worst pattern of invasion (WPOI), and lymph node involvement, with or without extranodal extension (ENE).

**Methodology:**

A retrospective, cross-sectional comparative analysis based on archival cases from the period of January 2021 to December 2023, in pathology department in a tertiary care hospital in central India. A total of 93 patients diagnosed with OSCC were included. All cases had available formalin-fixed, paraffin-embedded (FFPE) tissue blocks. Inclusion criteria: histologically confirmed OSCC with adequate archived material from all age groups of both sexes. Exclusion criteria: non-oral head and neck malignancies and inadequate tissue blocks.

**Results:**

A total of 21 cases with LVE were detected. All these LVE were identified by CD34 immunohistochemistry, out of which, 20 cases were also detected by H&E stain. CD34 identified only one additional case of LVE as compared to H&E stain. The difference was not statistically significant (Chi-square = 0.03, *p* = 0.86). Significant correlations were observed between LVE and lymph node involvement (*p* = 0.0008), tumor grade (*p* = 0.0007), perineural invasion (*p* = 0.00006), depth of invasion (*p* = 0.0004), and WPOI (*p* = 0.00001). No significant correlation of the LVE was found with tumor size (*p* = 0.1005) or ENE (*p* = 0.19).

**Conclusions:**

CD34 IHC does not significantly improve detection of LVE over conventional H&E staining in OSCC. However, LVE shows strong associations with several high-risk histopathological features, reaffirming its prognostic importance. In a properly processed sample with competent histopathologists, the routine use of CD34 may not be necessary for detecting LVE in cases of OSCC.

*Trial registration*: This study is registered with the Clinical Trials Registry of India (ICMR-NIMS). The registration number is CTRI/2024/04/065233.

**Supplementary Information:**

The online version contains supplementary material available at 10.1007/s12672-025-03633-6.

## Introduction

The 16th most prevalent cancer in the world is oral cancer. India tops the list of the highest incidence of oral cancer in both men and women [1]. Squamous cell carcinoma is the most common histological type, and surgical resection is the primary mode of treatment [2]. Chewing tobacco and smoking are the most common etiological factors [3]. These cancers exhibit a tendency for recurrence after initial surgical treatment [4]. Such recurrences significantly impact patients’ morbidity and mortality as well as increase the cost of treatment [5]. Understanding and mitigating the pattern of repeated recurrences in oral malignancy is crucial for optimizing patient care and outcomes. To reduce morbidity and mortality rates in oral cancer patients, early diagnosis, assessment of behavior, and prognosis are vital.

The American Joint Committee on Cancer (AJCC), eighth edition, takes into consideration the tumor size, local lymph node status, and distant metastatic spread as the parameters for staging the oral cancers [6]. Other histological parameters like tumor grade, lymphovascular and perineural invasion by tumor cells, and worst pattern of invasion (WPOI), although not part of the staging system, influence the prognosis and treatment outcome of a patient [7, 8]. These parameters, including lymphovascular emboli (LVE), are used to assess outcome, recurrence, and overall survival in patients with oral cancer.

The presence of LVE is an important prognostic factor predicting the recurrence of the disease [9]. Patients with LVE should be considered for further adjuvant therapy post-surgical excision of the tumor. Adjuvant radiotherapy and/or chemotherapy to patients with LVE have resulted in decreased recurrence rates [10]. While hematoxylin and eosin (H&E) staining is a standard method in pathology and provides a general overview of tissue architecture. Given the prognostic importance of the correct identification of lymphovascular emboli, specific staining techniques should be employed to distinguish lymphovascular emboli from artifact. CD34 immunostaining offers a specific advantage in detecting vascular structures, thus making it easier to identify lymphovascular emboli [11]. 

This study takes into consideration the role of CD34 in identifying LVE in all cases of oral cancer, especially those with negative LVE by H&E and lymph node negative status.

## Aim and objectives

The primary objectives of the study were:

1. To evaluate and compare the sensitivity and specificity of CD34 immunohistochemistry staining and conventional hematoxylin and eosin (H&E) staining in identifying lymphovascular emboli in oral squamous cell carcinoma cases.
2. To investigate the degree of concordance between CD34 immunohistochemistry staining and H&E staining results in identifying lymphovascular emboli, exploring instances of agreement and discrepancy.

The secondary objective of the study was to correlate the presence of lymphovascular emboli identified by CD34 staining and H&E staining with other prognostic indicators such as tumor stage, grade, perineural invasion, worst pattern of invasion (WPOI), and lymph node involvement, with or without extranodal extension (ENE).

## Methods and material

This study has employed a retrospective comparative analysis design, involving the examination of all cases of oral squamous cell carcinoma from the period of July 2021 to July 2024.

### Inclusion criteria

All the archived cases of oral squamous cell carcinoma, whose formalin-fixed paraffin-embedded tissue blocks were available in the department of pathology. The oral cavity subsites included were tongue, buccal mucosa & alveolus. The histological subtype included was squamous cell carcinoma.

### Exclusion criteria

Malignancy in the head & neck region apart from those involving tongue, buccal mucosa & alveolus (oral cavity), or any case whose blocks were not available in the archive, were not included in the study. Histological subtype other than squamous cell carcinoma were not included.

CD34 immunohistochemistry staining and conventional hematoxylin and eosin (H&E) staining have been performed on sections from each tissue block according to standard protocols. The monoclonal anti-CD34 antibody, clone QBEnd/10, and the Himedia’s Harris Hematoxylin and Eosin stain solution (2%) from Qualigens were used. Appropriate external control was used for H&E stain, while the endothelial lining of the normal vasculature of the oral mucosa acted as internal control for CD34. The histological slides were evaluated by two competent histopathologists. Lymphovascular emboli (LVE) positivity was defined as the presence of tumor cell aggregates within an endothelial-lined space. The statistical analysis was done by using descriptive and inferential statistics using Chi-square, and the software used in the analysis was SPSS 27.0 version and GraphPad 7.0 version. The p value of < 0.05 is considered the level of significance.

## Observations and results

### Population characteristics

**Fig. 1:**
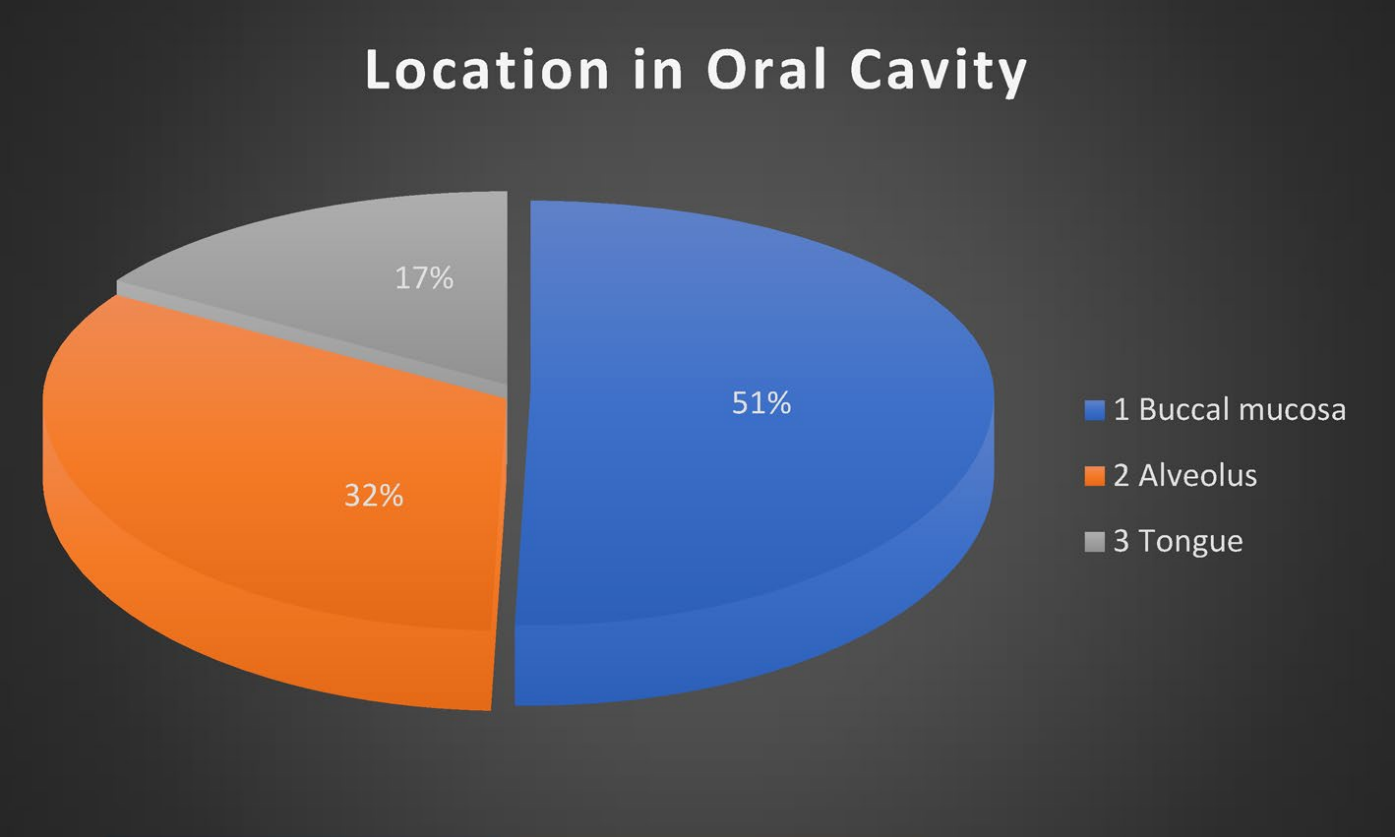
Tumor distribution as per location in the oral cavity

**Fig. 2:**
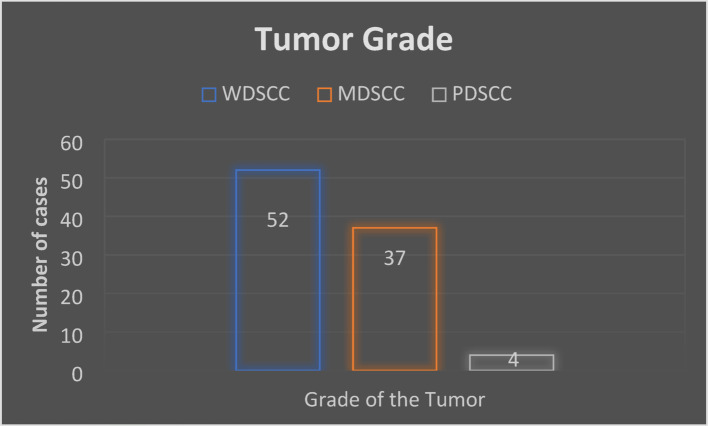
Distribution of patients according to grade of the tumor

Out of the 93 patients, most were in the age group of 40–50 years, with the mean age being 49 years, and the majority being male patients (57, 61%). The most commonly affected subsite was the buccal mucosa (47, 51%), followed by the alveolus (30, 32%) and lastly the tongue (16, 17%). The most common tumor stage was T3 (47, 51%), followed by T2 (26, 28%), T1 (14, 15%), and T4 (6, 6%). (Fig. 1) Lymph node involvement was present in 60 patients (65%), and extranodal extension (ENE) was seen in 5 patients (5%). Regarding tumor grade, well-differentiated squamous cell carcinoma (WDSCC) was the most prevalent (52, 56%), followed by moderately differentiated squamous cell carcinoma (MDSCC) (37, 40%) and poorly differentiated squamous cell carcinoma (PDSCC) (4, 4%). (Fig. 2) Perineural invasion (PNI) was found in 53 patients (57%).

**Fig. 3:**
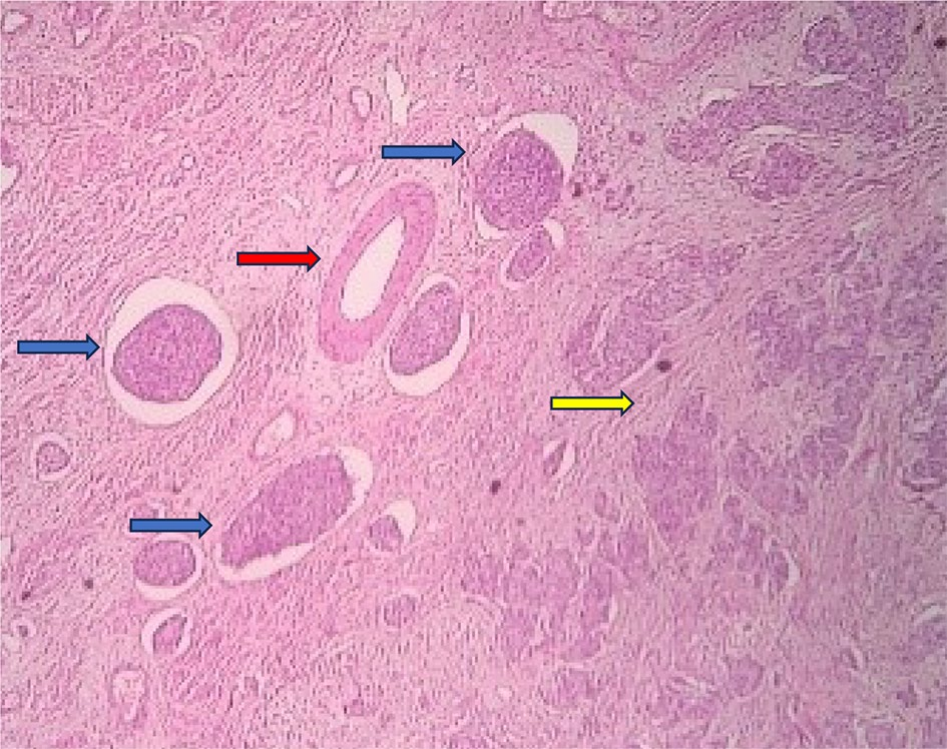
H & E stained section showing invasive squamous cell carcinoma (yellow arrow), vein (red arrow) and numerous LVI (blue arrow). [100X magnification]

**Fig. 4:**
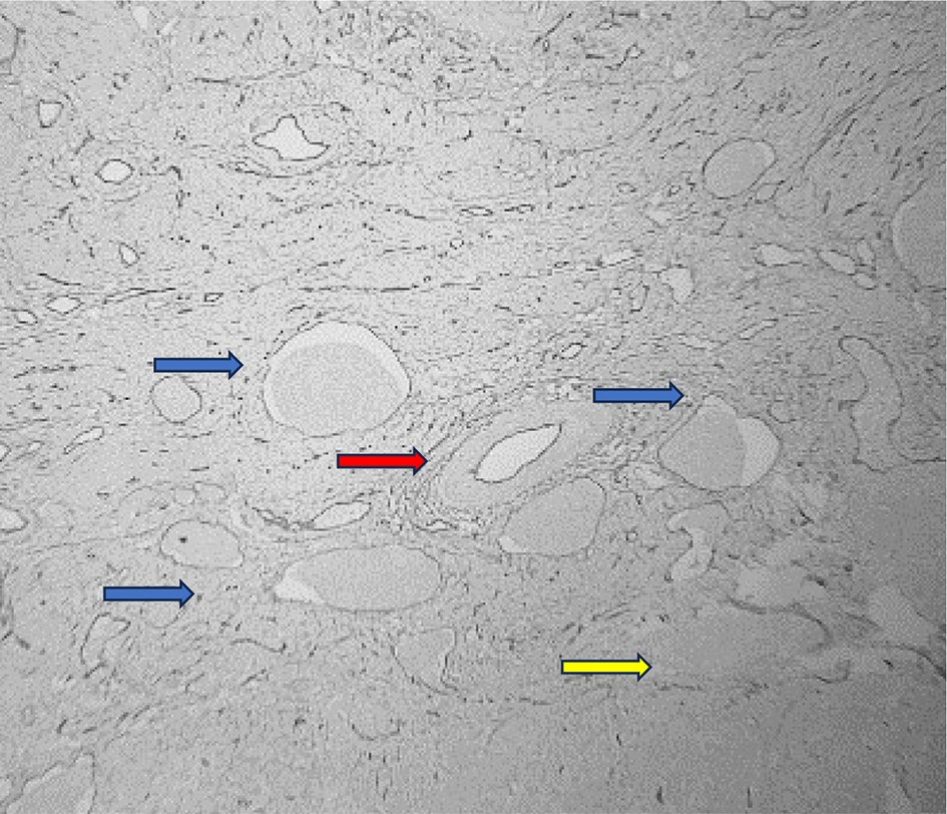
CD34 stained section showing membranous staining of the endothelial lining of the vein (red arrow). Numerous LVI represented by tumor cell deposits surrounded by CD34 positive endothelial cells (blue arrow). The invasive squamous cell carcinoma is also seen (yellow arrow). [100X magnification]

**Fig. 5:**
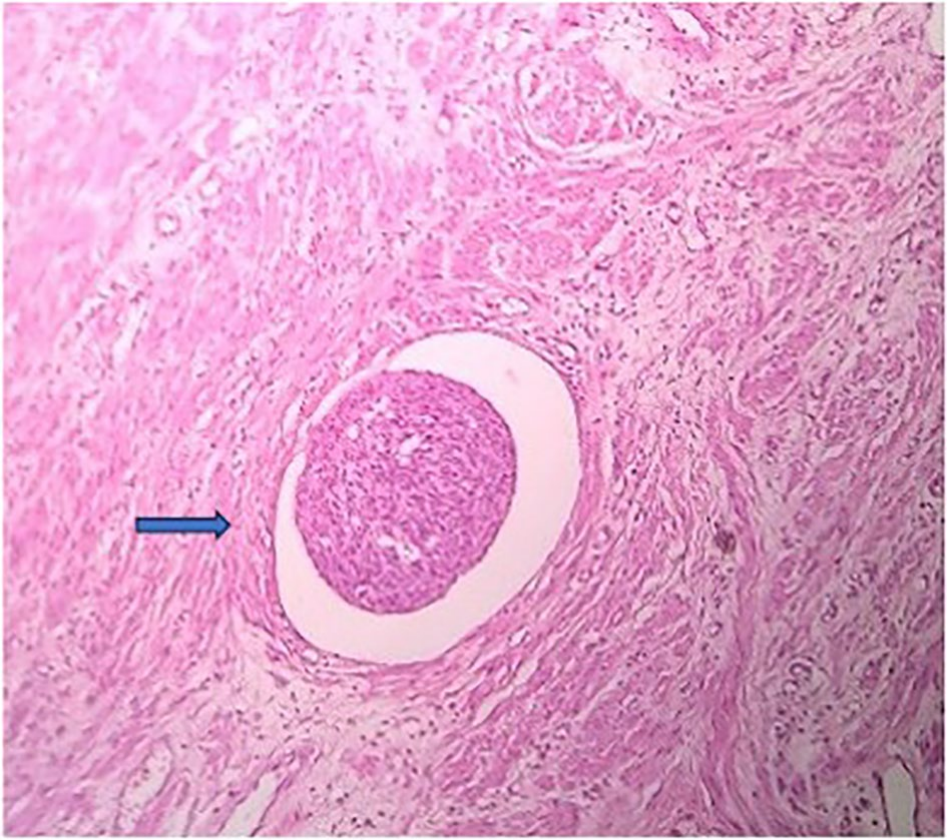
H & E stained section showing LVI (blue arrow). Note the endothelial cells are not easily recognised, creating a suspicion of artifactual retraction. [100X magnification]

**Fig. 6:**
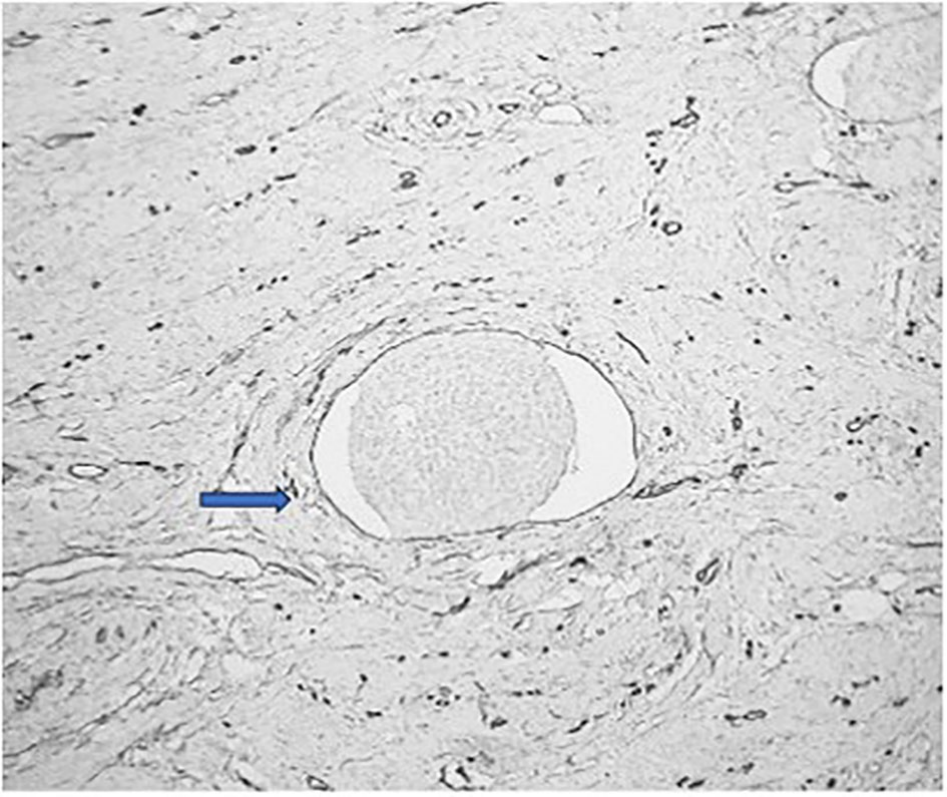
CD34 stained section highlighting the endothelial cell lining surrounding the tumor cells, confirming LVI (blue arrow). [100X magnification]

Among the study groups, a total of 21 cases with LVE were detected. All these LVE were identified by CD34 immunohistochemistry, out of which, 20 cases were also detected by H&E stain. In only one case was the LVE detected by CD34, which was not identified by the regular H&E stain. The chi-square statistic was 0.03. The p-value was 0.86 (*p* > 0.05), which is not significant. (Table 1) This suggests that there is not enough evidence to conclude that CD34 immunohistochemistry staining significantly improves the identification of lymphovascular emboli and aids in better differentiation from retraction artifacts compared to conventional H&E staining. (Figures 3, 4, 5 and 6)

**Table 1:**
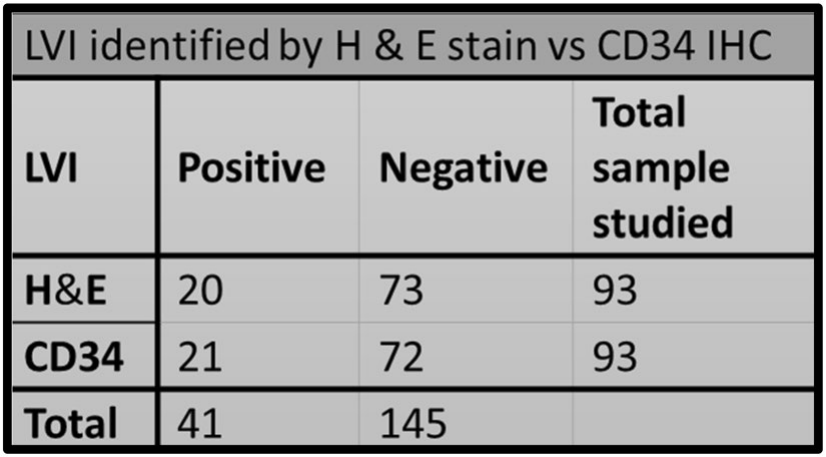
Detection of LVI using H&E and CD34

#### Association of LVE with other histological parameters

(Table 2)

**Table 2:**
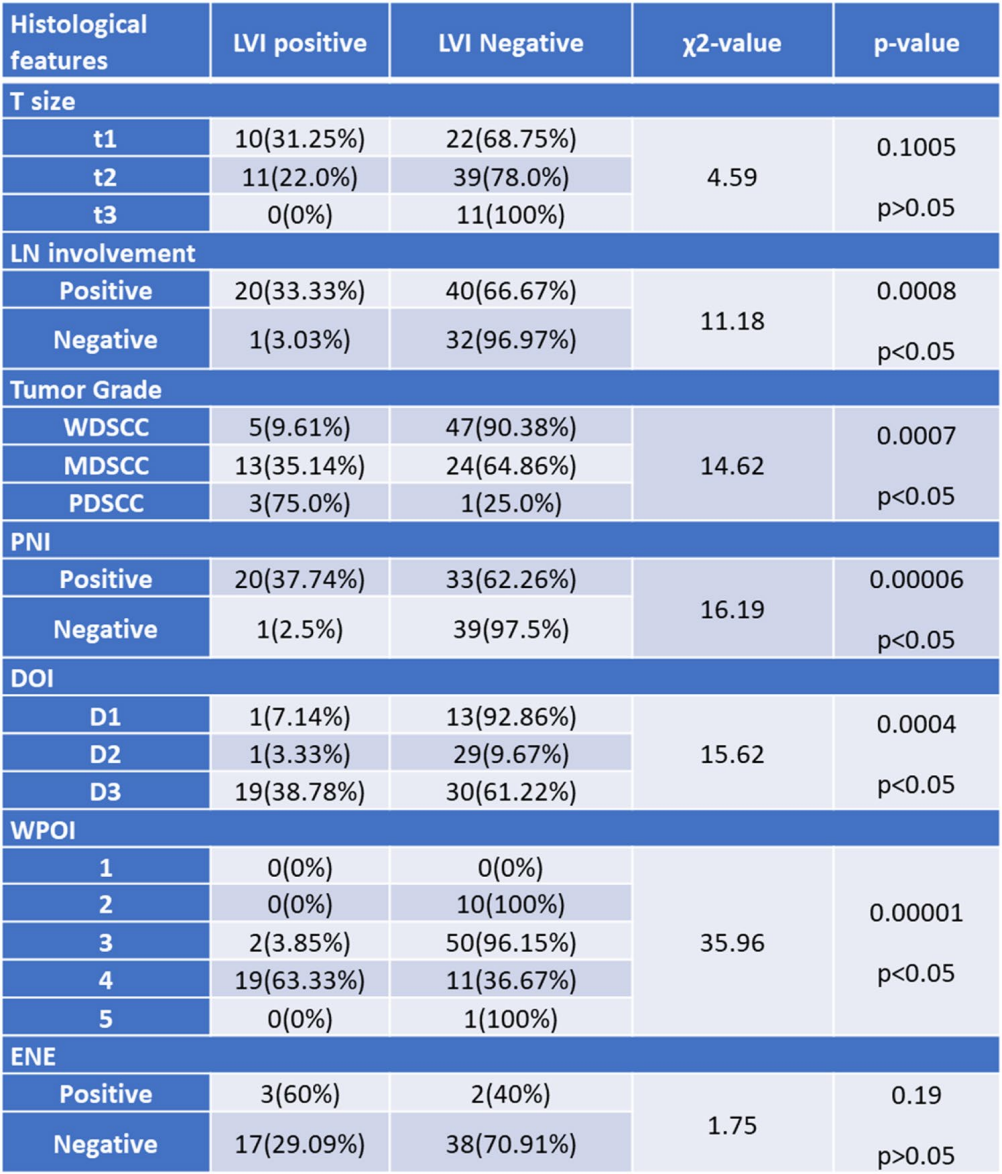
Correlation of LVI with other histological parameters

#### Association of lymph node (LN) involvement and LVE

In the 60 cases that showed LN involvement, 20 (33.33%) had LVE positive, whereas 40 (66.67%) had LVE negative. In the 33 cases where LN involvement was not seen, LVE was detected in only one (3.03%) case, while the rest of the 32 (96.97%) cases were negative for LVE. The chi-square statistic was 11.18. The p-value was 0.0008. The result is significant at *p* < 0.05.

#### Association of tumor grade and LVE

Five (9.61%) out of 52 WDSCC showed LVE positivity, while 47 (90.38%) were negative. For MDSCC, 13 (35.14%) out of 37 were LVE positive and 24 (64.86%) were negative. For PDSCC, three (75%) out of four were LVE positive and one (25%) was negative. The chi-square statistic was 14.62. The p-value was 0.0007. The result is significant at *p* < 0.05.

#### Association of perineural invasion (PNI) and LVE

20 (37.74%) cases had both LVE and PNI, while 39 (97.5%) cases had neither. 33 (62.26%) had PNI but did not show LVE. The chi-square statistic was 16.19. The p-value was 0.00006. The result is significant at *p* < 0.05.

#### Association of depth of invasion (DOI) and LVE

19 (38.78%) cases with DOI > 1.0 cm displayed LVE, whereas only 1 (7.14%) and 1 (3.33%) with DOI of less than or equal to 0.5 cm and between 0.5 and 1.0 cm had LVE. The chi-square statistic was 15.62. The p-value was 0.0004. The result is significant at *p* < 0.05.

#### Association of worst pattern of invasion (WPOI) and LVE

**Fig. 7:**
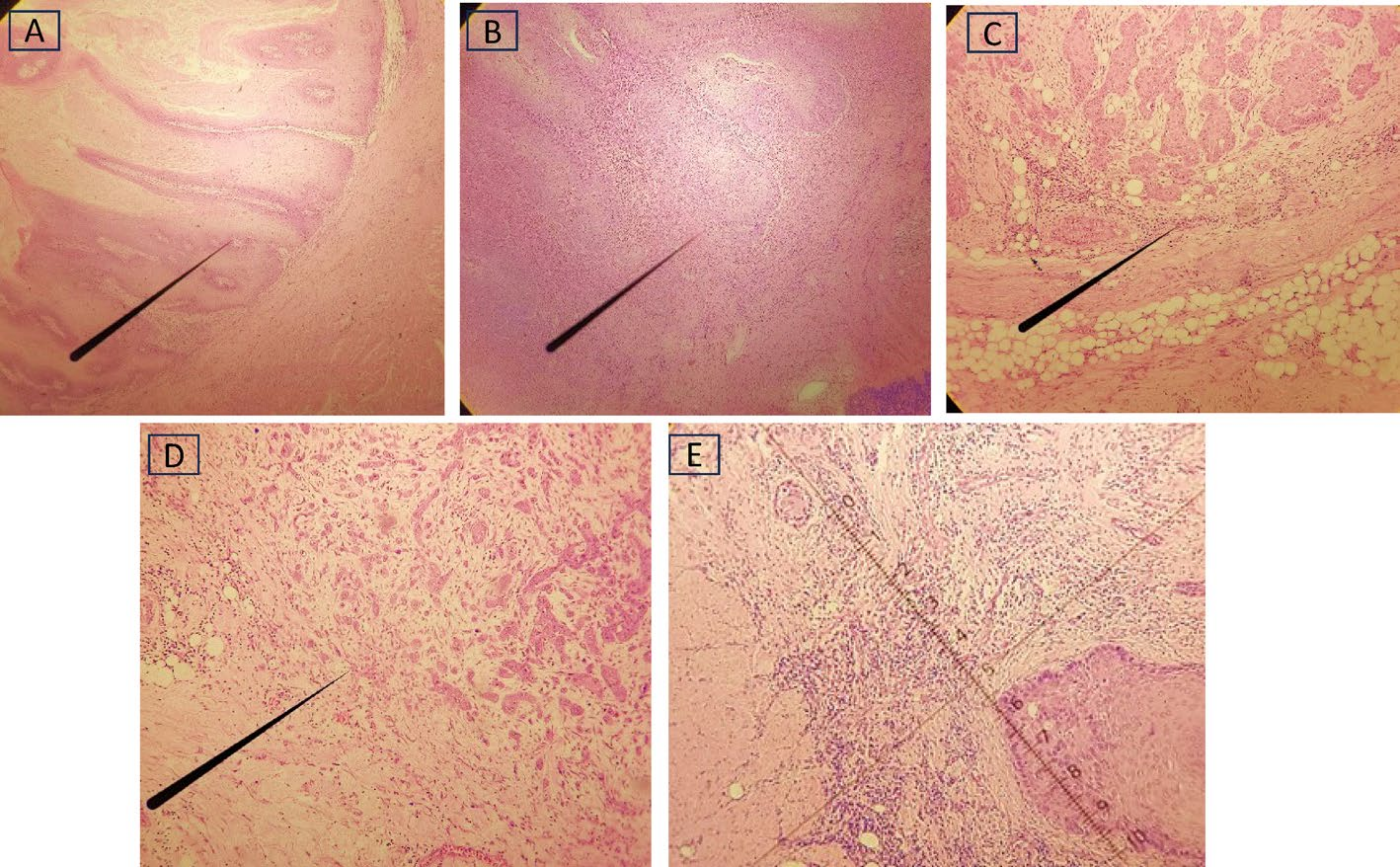
Worst Pattern of Invasion (WPOI). Pattern I (A); Pattern II (B); Pattern III (C); Pattern IV (D); Pattern V (E). [H & E; 40X magnification]

19 (63.33%) of type 4 WPOI had LVE, whereas 50 (96.15%) of type 3 did not have LVE. (Fig. 7) The chi-square statistic was 35.96. The p-value was 0.00001. The result is significant at *p* < 0.05.

#### Association of tumor size (T) and LVE

In the current study, none of the tumors larger than 4 cm showed LVE, whereas 10 (31.25%) tumors with size ≤ 2 cm and 11 (22.0%) tumors with sizes between 2 and 4 cm had LVE. The chi-square statistic was 4.59. The p-value was 0.1005 (*p* > 0.05). The result is not significant.

#### Association of extra-nodal extension (ENE) and LVE

Although LN involvement showed a significant association with LVE, extra-nodal extension did not, with the chi-square statistic value of 1.75 and the p-value of 0.19 (*p* > 0.05).

## Discussion

There are several microscopic features that affect the prognosis of oral cancer that are not included in the AJCC TNM staging. The features have been shown to affect the prognosis of the patients. Many patients have a combination of two or more adverse histopathological features. The study showed a positive correlation of LVE with LN involvement, tumor grade, PNI, WPOI, and DOI, whereas no positive correlation was observed with tumor size and ENE. The study showed that CD34 immunohistostaining to diagnose lymphovascular emboli is unnecessary in practical settings, and H&E staining may be sufficient to diagnose lymphovascular emboli.

### LVE detection by IHC CD34 AND H&E

The current study explored the detection of lymphovascular emboli (LVE) using CD34 immunohistochemistry (IHC) compared to the conventional Hematoxylin & Eosin (H&E) staining method. The study found that CD34 detected LVE in 21 cases, while H&E detected it in 20 cases. The chi-square statistic of 0.03 and a p-value of 0.86 suggest no significant difference between the two methods in LVE detection (*p* > 0.05). This contrasts sharply with findings from O’Donnell RK et al. [12], where a triple-stain technique using cytokeratin, CD34, and podoplanin identified LVE in 102 cases compared to only 13 cases detected by H&E, with a highly significant p-value of < 0.001. The profound difference observed in the O’Donnell study, particularly in intratumoral LVE, highlights the potential limitations of conventional H&E staining and suggests that IHC, especially with combined markers, could offer superior sensitivity. Similarly, Mascitti M et al. [13] pointed out that LVE detection variability in oral squamous cell carcinoma (OSCC) can be influenced by multiple factors, including the number of tumor sections examined and the staining method used. Their study highlighted a prevalence range of 3% to 90% for LVE in OSCC, underscoring the challenges in achieving consistent detection. The current study’s minimal difference between CD34 and H&E (one additional case detected by CD34) suggests that while CD34 IHC may offer slight advantages, its impact in routine practice may be less pronounced unless combined with additional markers, as seen in the O’Donnell study.

### Correlation of LVE with tumor size (T stage)

The current study found no significant correlation between tumor size (T stage) and LVE, with the chi-square statistic at 4.59 and a p-value of 0.1005 (*p* > 0.05). This is contrary to the findings of Huang Q et al. [14], where a significant association between positive LVE and T stage was observed (Chi-square = 15.19, *p* = 0.002). In the current study, LVE was found in 31.25% of tumors ≤ 2 cm and 22.0% of tumors between 2 and 4 cm, but none in tumors larger than 4 cm. This suggests that while smaller tumors may present with LVE, the presence of LVE does not necessarily increase with larger tumor sizes in this cohort, which could be due to the sample characteristics or other biological factors not explored in this study.

### Correlation of LVE with lymph node involvement

A significant association between LVE and lymph node (LN) involvement was observed in the current study (Chi-square = 11.18, *p* = 0.0008). This finding aligns with the results from Huang S et al. [15] and Huang Q et al. [14], who also reported a significant correlation between positive LVE and LN metastasis. Specifically, Huang S et al. found that 62.6% of cases with positive LVE had LN metastasis (*p* < 0.00001), while Huang Q et al. reported a significant Chi-square of 25.83 (*p* < 0.001). The current study’s findings reinforce the role of LVE as a critical predictor of LN metastasis, emphasizing the importance of LVE detection in the clinical management of oral cancer patients.

### Correlation of LVE with tumor grade

The current study demonstrated a significant correlation between LVE and tumor grade (Chi-square = 14.62, *p* = 0.0007). This is consistent with the findings of Huang Q et al. [13], who reported a significant association between LVE and histological grade (Chi-square = 5.89, *p* = 0.117). In the current study, LVE positivity was highest in poorly differentiated squamous cell carcinoma (PDSCC) (75%) compared to moderately differentiated (MDSCC) (35.14%) and well-differentiated squamous cell carcinoma (WDSCC) (9.61%). This trend suggests that higher tumor grades are more likely to exhibit LVE, indicating a more aggressive tumor phenotype. However, Khan S et al. [16] reported no significant difference between LVE and histological grading, which may be due to different study populations or methodologies.

### Correlation of LVE with WPOI

The current study found a significant correlation between LVE and worst pattern of invasion (WPOI) (Chi-square = 35.96, *p* = 0.00001), with 63.33% of WPOI type 4 cases showing LVE compared to 3.85% in WPOI type 3. This result is in line with Yasuda M et al. [17], who also observed a significant association between LVE and WPOI 4 (*P* = 0.014). The strong correlation in the current study suggests that WPOI could serve as an important morphological marker in predicting the presence of LVE, further supporting its inclusion in routine histopathological assessments for oral cancer.

### Correlation of LVE with PNI

The current study demonstrated a significant association between LVE and perineural invasion (PNI) (Chi-square = 16.19, *p* = 0.00006), with 37.74% of cases showing both LVE and PNI. This finding aligns with the observations of Huang Q et al. [14], where both LVE and PNI were significantly correlated with overall survival (*p* = 0.001 for LVE). Similarly, Mascitti M et al. [13] highlighted the relationship between LVE, PNI, and other aggressive histological features, suggesting a cluster of high-risk factors that collectively contribute to poor prognosis. The current study’s results confirm the interplay between LVE and PNI, underscoring the need for their combined assessment in evaluating the aggressiveness of oral cancer.

*Correlation of LVE with DOI*: In the current study, a significant correlation was found between LVE and depth of invasion (DOI) (Chi-square = 15.62, *p* = 0.0004). LVE was detected in 38.78% of cases with DOI > 1.0 cm, compared to much lower rates in tumors with lesser DOI.This finding is consistent with Moore AE et al. [18], who reported that LVE was a significant predictor of cervical node metastasis when controlled for DOI (OR: 3.42, *p* < 0.001). The current study’s results suggest that tumors with greater DOI are more likely to exhibit LVE, reinforcing the value of DOI as a prognostic marker in oral cancer.

Each of the above histological parameters are recognised as poor prognostic factors, individually. The combined presence of LVE with a higher tumor grade, greater DOI, presence of PNI, higher WPOI, and lymph node involvement, suggest a higher likelihood of metastases extension or recurrence of the tumor. Thus, their combined presence often leads to more aggressive treatment protocol in the form of postoperative chemotherapy and/or radiotherapy. A more intense surveillance may be suggested to monitor for metastases or recurrence in such cases [14, 19].

## Conclusion

The study showed that using the immunohistochemistry marker for endothelial cells—CD34—does not improve the diagnostic efficacy for lymphovascular emboli over careful examination of the routine Hematoxyline & Eosin-stained sections.

The combined presence of lymphovascular emboli with a higher tumor grade, greater depth of invasion, presence of perineural invasion, higher worst pattern of invasion, and lymph node involvement, suggest a higher likelihood of metastases extension or recurrence of the tumor. Their combined presence suggests a higher likelihood of metastases extension or recurrence of the tumor.

Thus, a combined comprehensive evaluation of several clinical and histopathological factors might be a more definitive way to predict the outcome and often helping in planning a more aggressive treatment protocol and a more intense surveillance program to monitor for metastases or tumor recurrence.

## Strengths and limitations of this study

### Strengths

The study utilized a dual staining protocol (H&E and CD34 IHC), allowing direct methodological comparison within the same tissue samples.

Histopathological evaluation was performed by two independent, experienced pathologists, reducing inter-observer variability.

The retrospective design enabled analysis of real-world, archived clinical material with a practical, cost-effective approach.

### Limitations

Being a single-centre study limits external validity and may not capture institutional or regional variations in specimen processing or interpretation. The relatively small sample size also limits the range of patients with different environmental and genetic influences.

Lack of association of the presence of lymphovascular emboli and other histological parameters with the long-term follow-up & clinical outcome data, such as recurrence or disease-free survival, limits prognostic interpretation.

## Supplementary Information

Below is the link to the electronic supplementary material.

Supplementary Material 1

## Data Availability

Data is provided within the manuscript and the supplementary information files.
